# Multicystic nephroma masquerading as hydatid cyst: a diagnostic challenge

**DOI:** 10.1186/s12894-017-0208-4

**Published:** 2017-03-11

**Authors:** Abdelmoneim E. M. Kheir, Aziza M. Elnaeema, Sara M. A. Gafer, Sawsan A. Mohammed, Mustafa E. Bahar

**Affiliations:** 1 Department of Paediatrics and Child Health, Faculty of Medicine, University of Khartoum and Soba University Hospital, P.O. Box 102, Khartoum, Sudan; 2 Paediatric Surgeon and Paediatric Urologist, Soba University Hospital, Ahfad University for Women, Omdurman, Sudan; 3 Department of Paediatrics, Soba University Hospital, Khartoum, Sudan; 4 Department of Histopathology, Soba University Hospital, Khartoum, Sudan; 5 Department of Radiology, Soba University Hospital, Khartoum, Sudan

**Keywords:** Multicystic nephroma, Hydatid cyst, Benign, Sudan, Case report

## Abstract

**Background:**

Multicystic nephroma is an uncommon, non-familial renal neoplasm that is usually benign. About 200 cases of this lesion have been described in the literature.

**Case presentation:**

We report on a Sudanese child who presented at the age of two and a half years with an abdominal mass, clinical and radiological features favored the diagnosis of hydatid cyst which is endemic in this African tropical country, and the diagnosis of multicystic nephroma was only possible after histopathological examination.

**Conclusion:**

Multicystic nephroma is a rare benign tumour with an excellent prognosis. Clinical and radiological differentiation of multicystic nephroma from hydatid cyst is difficult. Thus, histopathological examination of the surgical specimens seems to be the only feasible method of making the correct diagnosis.

## Background

Multicystic nephroma (MCN) is a rare, non-familial renal tumour, that has a benign nature. About 200 cases of this lesion have been reported so far [1]. Different names have been used to describe this renal mass, including solitary multilocular cyst, multilocular renal cyst, renal cystadenoma, cystic renal hamartoma and partial polycystic kidney. Due to similarities in age, sex and histochemical profile, adult cystic nephroma is now classified within this group of mixed epithelial and stromal tumours, and the world health organization (WHO) renal tumour subcommittee recommended using the term mixed epithelial and stromal tumour family for both entities [2]. As opposed to adult cystic nephroma, paediatric cystic nephroma is now regarded a separate entity with specific DICER1 mutations [3]. MCN can be seen in both infants and adults. Seventy-three percent of the patients are males and aged between 2 and 4 years [4].

The pathogenesis of MCN remains unclear, [5] thus its origin is designated as being dysplastic/hamartomatous/neoplastic. Histologic features include: cysts lined by flat, cuboidal, or hobnail epithelium and septa variably lined by fibrous and/or ovarian-like stroma. These histological features are quite unique, however confusion does occur with other cystic renal tumours, especially cystic renal cell carcinoma which can lead to conflict in the treatment of this lesion [6].

We describe a Sudanese child who presented at the age of two and a half years with an abdominal mass, clinical and radiological features favored the diagnosis of hydatid cyst and the diagnosis of multicystic nephroma was only possible after histopathological examination. To our knowledge this is the first case report of multicystic nephroma masquerading as hydatid cyst from a tropical country in Africa.

## Case presentation

**Fig. 1:**
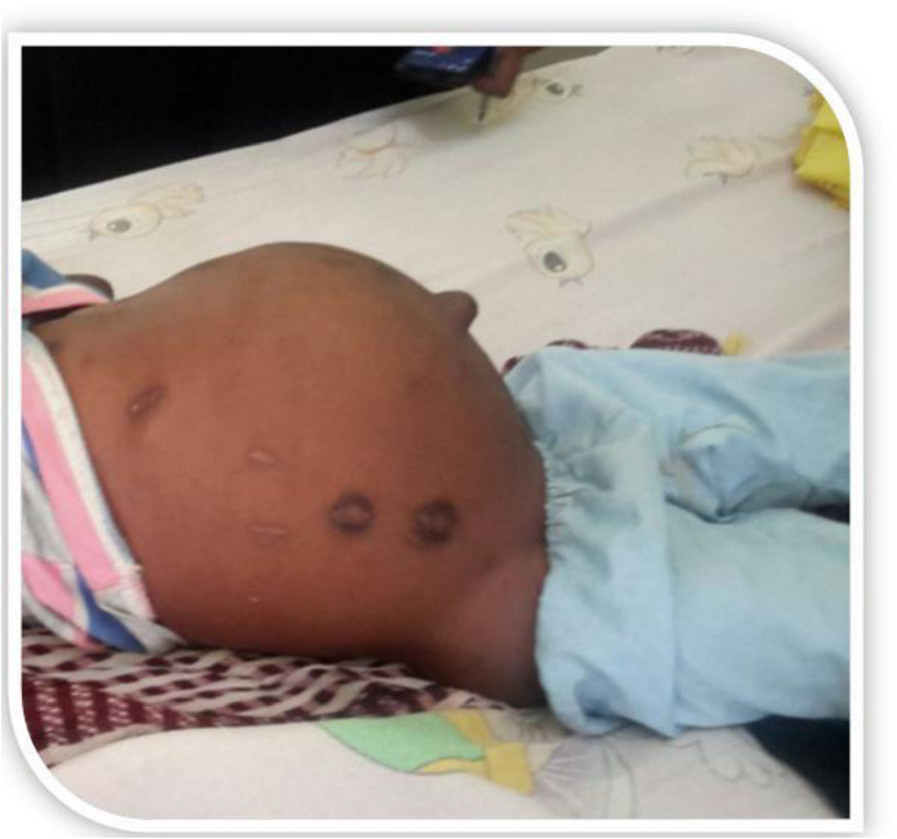
Shows gross abdominal distension, cautery *marks* and umbilical hemia

A two and a half year old male child from Darfur province, west of Sudan was brought by his parents because of abdominal distension for 8 months prior to admission. The distension was associated with mild pain and no symptoms related to the urinary or gastrointestinal systems. His past medical history was unremarkable apart from poor socioeconomic status and contact with livestock and sheepdogs. Physical examination revealed a slightly pale child with no jaundice and no lymphadenopathy. Abdomen was grossly distended with bulging of the right flank, there was umbilical hernia. There were cautery marks, striae, and superficial veins (Fig. 1). Superficially abdomen was tense with palpable right sided abdominal mass extending from the right renal angel to the umbilicus about (15 × 25 cm), no hepatosplenomegaly and negative shifting dullness. His vital signs including blood pressure were normal.

Investigations performed included a full blood count which showed nutritional anaemia, ESR 57 mm/h. His renal and liver function tests were entirely normal. Urine analysis was also normal. CXR was normal.

**Fig. 2:**
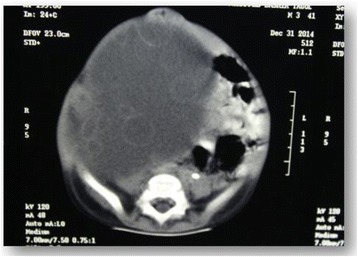
CT abdomen shows a *large* cystic mass arising from the right kidney

**Fig. 3:**
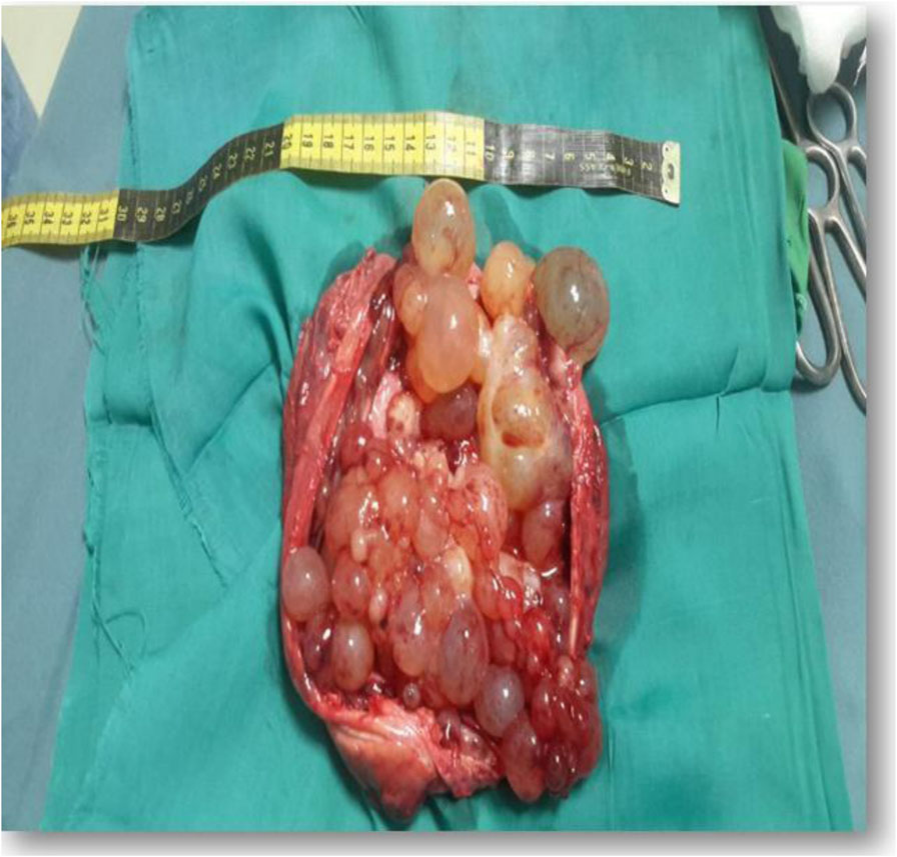
Shows a *large* cystic mass that contains numerous cysts

**Fig. 4:**
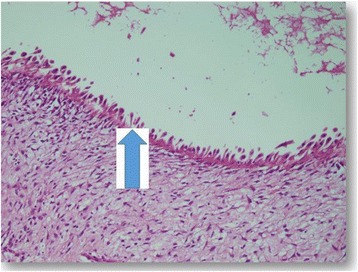
Shows hobnail pattern (*arrowed*)

The first abdominal ultrasound revealed a huge right cystic renal mass, containing small daughter cysts arranged eccentrically as well as few septations with normal left kidney. Findings were compatible with right renal embryonic multi cystic tumor. A repeat ultrasound in a different setup showed the same findings with a strong possibility of hydatid renal disease. Computed tomography (CT) of the chest was normal and CT abdomen showed a large cystic mass arising from the right kidney consistent with hydatid cyst (Fig. 2). The paediatric surgeon was consulted who requested a dynamic renal scan that showed a nonfunctioning right kidney with features of parenchymal effacement. The suspicion for hydatid disease was high based on the radiological findings of a large cystic mass, the geographical prevalence and patient risk factors for hydatid cyst. The child was commenced on Albendazole (antihelminthic drug) and a radical right sided nephrectomy was performed 4 weeks later and the cyst ruptured during the operation (Fig. 3), the dimensions of the mass was 7×13 cm. Histopathological examination showed multiple cysts separated from renal tissue by fibrous tissue, the cysts were lined by flattened to columnar epithelium, some showing hobnail pattern (Fig. 4). The renal tissue was composed of hyalinized glomeruli, atrophied tubules and mixed inflammatory cellular infiltration. All was consistent with multicystic nephroma. The child had a benign postoperative course and was discharged home in good condition with a view for follow up in outpatient clinic.

## Discussion

The first case of MCN in the literature was reported by Edmund et al. as cystic nephroma of the kidney in 1892 [7]. In 1956, Boggs and Kimmelstiel first proposed the true neoplastic nature of the lesions in a case report, suggesting the term benign multilocular cystic nephroma for this condition [8].

MCN is diagnosed by set of criteria as suggested by Powell et al. in 1951 [9] and later modified in 1956 [8]. The diagnostic criteria of cystic nephroma established by Joshi and Beckwith et al. [10] are used widely and include the following: multilocular, solitary, unilateral, noncommunication between the renal pelvis and the cystic lesion, a definite lining of epithelium on the loculi, no nephron in the interlobular septa, and normal residual renal tissue. Our case meets these criteria.

Our case was a two and a half year old male child with unilateral MCN, and this is similar to what is reported in the literature [4], however bilateral cases also have been reported [2]. The usual presentation of MCN is a benign clinical course, asymptomatic abdominal mass, with non-specific symptoms as abdominal pain, hematuria, and urinary tract infection [11]. The main complaint in our case was abdominal mass and pain without haematuria. Haematuria can be seen in all age groups and is thought to be due to extension of tumor into the renal pelvis [12]. Rarely presentation can sometimes be with severe colicky abdominal pain due to spontaneous rupture of the cyst [13].

The background history of our case was poor socioeconomic status, poor sanitation and contact with livestock and sheepdogs, all these, together with the radiological findings, favored the diagnosis of hydatid cyst (Echinococcosis) . Cystic echinococcosis is caused by infection with the larvae of Echinococcus granulosus. Areas of the world with noted prevalence are rural regions of Africa, southern Europe, Asia, the Middle East, Central and South America and is principally maintained in a dog–sheep–dog cycle, [14]. Serological tests were not done for our case because they aren’t available.

At present there is no reliable clinical or radiographic means to differentiate cystic nephroma from other cystic renal disease in children [15]. The non-specific clinical findings and the poor contribution of imaging studies make the exact preoperative distinction from other cystic renal neoplasia difficult and as a result

histopathological examination from a resected specimen seems to be the only feasible method of making the correct diagnosis [5].

Because preoperative diagnosis is difficult to achieve and multicystic renal cell carcinoma is suspected, radical nephrectomy is the standard treatment of choice. Our case had a radical right nephrectomy as the diagnosis was uncertain and the dynamic renal scan showed a nonfunctioning right kidney. In cases of solitary, localized, unilateral lesions less than 4 cm with frozen section proven diagnosis, nephron-sparing surgery is advocated [16]. The prognosis is usually good for MCN and surgical excision is curative, however, these cases should be followed up because three cases of local recurrence have been reported [17].

## Conclusion

MCN is a rare benign tumour which has a good prognosis. Clinical and radiological differential diagnosis of MCN from hydatid cyst is difficult. Thus, histopathological examination of the surgical specimens seems to be the only feasible method of making the correct diagnosis.

## Abbreviations

CT: Computed tomography
MCN: Multicystic nephroma
WHO: World Health Organization
